# Supplementary material for: Chromothripsis during telomere crisis is independent of NHEJ, and consistent with a replicative origin
Source: Genome Res. 2019 May;29(5):737–49. doi: 10.1101/gr.240705.118 (PMC6499312; doi:10.1101/gr.240705.118)
Supplement: Supplemental Material [file supp_gr.240705.118_Supplemental_file_1.zip › contigs/annotated_contigs/DB37/contig.2.DB37_length_661_mean_cov_7.65809379728.docx]

**DB37_length_661_mean_cov_7.65809379728**

```
CTCGGCCCCAGCGCACGCCGTGTTCAGCGCAGTCTCGCCCTGGCTGGTGCGCAGGCCCACGTCGGCGCCATGCTCCAGGTACAGAGCCA
 >chr17:42253887-42254301 - E=2e-237 p=1e-02
CATGTTGCTCCAGGCCGCGCGCCGCCGCCACGTGCAGGGGCGTCTCCTGGCTCTCGCCTGCTGCCAGGTTCACCGTCGCTCCTGCTTCC

AGCAGCAACTTGGCGCACCTGGGGACAAGAGAGCAAGGTCCTGAGGAAAATCCTGAAGGCTTGGGACCCCTTCAGCTTGCTGTGACACT

CCCTTGCCTTTCCACCCCTCTCAGCTTGAGGTCCTACCTGGTGTCCTCCATGAGAATCATACAAGCCCCGCTACAGACAACAGAATTGG

AAGGTCCTGAGAGCTACCAGAAGCATCTTCAGTTTTCATATGAAAAACTTAAGCCCAG|CCGAGACTGTGT|GAGGCCGAGACTGTGAG
                                                                       >chr17:42155718-
CGCCATGGCCTTGCTGTCGGTGGGGGCCGAGCAGGCCCAGGCTGCGGCAGCCCGGGAACACAGCCCCAGGTAAGCAGCAACCCGCACCA
42155953 - E=1e-129
CCTCTCTGCCCTCACCCCTGCTGGTGCGCCCCACTCAATACCCTCCCCTGTGCTTGCCCCACAGGCCGGCAGAGGAGCCCATGGAGCAG

GAGCCTGCCCTGTGACGCCCCGGCCCCCATCCCTCTGGGC
```
